# Investigating the efficiency of dynamic vaccination by consolidating detecting errors and vaccine efficacy

**DOI:** 10.1038/s41598-022-12039-1

**Published:** 2022-05-17

**Authors:** Yuichi Tatsukawa, Md. Rajib Arefin, Shinobu Utsumi, Jun Tanimoto

**Affiliations:** 1 Interdisciplinary Graduate School of Engineering Sciences, Kyushu University, Kasuga-koen, Kasuga, Fukuoka 816-8580 Japan; 2 Department of Mathematics, University of Dhaka, Dhaka, 1000 Bangladesh; 3 Faculty of Engineering Sciences, Kyushu University, Kasuga-koen, Kasuga, Fukuoka 816-8580 Japan

**Keywords:** Computational biology and bioinformatics, Mathematics and computing

## Abstract

Vaccination, if available, is the best preventive measure against infectious diseases. It is, however, needed to prudently design vaccination strategies to successfully mitigate the disease spreading, especially in a time when vaccine scarcity is inevitable. Here we investigate a vaccination strategy on a scale-free network where susceptible individuals, who have social connections with infected people, are being detected and given vaccination before having any physical contact with the infected one. Nevertheless, detecting susceptible (also infected ones) may not be perfect due to the lack of information. Also, vaccines do not confer perfect immunity in reality. We incorporate these pragmatic hindrances in our analysis. We find that if vaccines are highly efficacious, and the detecting error is low, then it is possible to confine the disease spreading—by administering a less amount of vaccination—within a short period. In a situation where tracing susceptible seems difficult, then expanding the range for vaccination targets can be socially advantageous only if vaccines are effective enough. Our analysis further reveals that a more frequent screening for vaccination can reduce the effect of detecting errors. In the end, we present a link percolation-based analytic method to approximate the results of our simulation.

## Introduction

Infectious diseases have been encumbering us for a long, claiming lots of lives besides financial burdens, and therefore is regarded as one of the major threats to human society^1,2^. Due to the high mobility in this modern age, communicable diseases can rapidly spread throughout the world. The ongoing Covid-19 pandemic has already caused grave catastrophes to the global public health and is continuing to do so. The near past has experienced outbreaks of several infectious diseases among which influenza is the most circulated one that recurrently appears with new strains^3^. It is much needed for policymakers to comprehend the spreading trends as well as possible consequences of the disease so that they can undertake a better plan to control the disease diffusion. Mathematical modeling can incorporate diverse features of the disease dynamics and then suggest the best use of control strategies among several alternatives, analyzing the short- and long-term effects by considering several constraints of the society^4^. Since the seminal contribution of Kermack and Mckendrick^5^ in 1927, it has become a standard practice to analyze disease dynamics through compartment modeling to predict the possible consequences and to compare the effects of different management strategies^6^. Vaccination, if available, is the most effective strategy for suppressing the disease spreading since it directly removes the fraction of the host population (susceptible) into the immune class via successful immunization^7^. When vaccines are unavailable, other protocols such as quarantine and isolation^4,8,9^, social distancing^10–12^, wearing masks^13,14^, border restrictions^15,16^, etc. have been used as alternative options to control or delay the peak of the epidemic so that health authorities get time to develop vaccines to stop the epidemic. Even if vaccines are developed, owing to supply and demand constraints, there is a possibility of vaccine shortages (for instance, Ref.^17^ for Covid-19). Therefore, it is much needed to strategically design the vaccination campaign to efficiently mitigate the disease spreading with minimizing social costs.

It is well known that infectious diseases spread through physical contact between individuals who are connected by some social networks^18,19^. Over the last few years, it has become a common practice to model contact patterns^20–22^ on complex networks where each node corresponds to an individual, and links among nodes represent interactions among individuals^23^. On such networks, one can explore the disease dynamics through a susceptible-infected-recovered (SIR) process by grouping individuals according to their states. If a susceptible individual has contact with an infected one, then the former becomes infected with a probability $\beta$. The infected individuals recover at a rate $\gamma$ (day^−1^). In this work, with the aid of multi-agent simulation (MAS), we investigate such a process by dovetailing a ‘dynamic vaccination’ campaign on a scale-free network^24^ in which susceptible neighbors become vaccinated—before having any physical contact with the infected individual—to avoid the infection. However, due to the lack of information, errors can occur in tracing infected individuals or the neighbors of infected people, or both. Our model takes into account such detecting errors. The model further assumes vaccine’s imperfectness to comply with reality. In the latter case, we assume that if any susceptible neighbor of an infected person misses out on immunity from vaccination, that particular neighbor will not be given another chance to vaccinate during the epidemic period. A similar line of research can be found in Ref.^23^ where—on a multiplex network—susceptible neighbors of an infected agent get vaccinated with a probability prior to physical contact with an infected patient. The authors mapped the ‘dynamic vaccination’ model into link percolation^25,26^ and used a generating function^27^ framework to theoretically predict the steady-state behavior. The theoretical results were found to be in a nice agreement with that of agent-based stochastic simulations or multi-agent simulations. The basic difference between Ref.^23^ and the current framework is that the latter introduces the notion of several detecting errors (i.e., error in tracing infected agents, their neighbors, and both). In addition, we further take into account the provision of vaccine imperfectness to capture the reality. Percolation models of statistical physics have been extended into a flexible framework for predicting infectious disease dynamics^19,28,29^. Unlike other methods for networked populations, such as degree-based mean field approach for SIR process^30–32^ in which deriving an analytic expression for the epidemic threshold or the final epidemic size is not always easy, the link percolation framework can solve a large class of standard epidemiological models on networks, giving exact solutions for the final size of the epidemic, presence of an epidemic, and other quantities of interests^19^. This fact has motivated us to present a link percolation-based model—dovetailing imperfect vaccination and error in tracing susceptible neighbors—to compare results arising from the theory and stochastic simulations. In this regard, we particularly follow the methodology presented in Refs.^19,23,29^. The analytic procedure gives us an exact expression for the vaccination coverage as well as the final epidemic size. On the ‘error versus vaccine efficacy’ phase plane, the results, concerning vaccination coverage and the final size of the epidemic, arising from the theoretical equations are generally found to be slightly less than that of the agent-based stochastic simulations. We argue that such a discrepancy appears due to the presence of super-hub agents in agent-based simulations.

The rest of the paper is organized as follows. The second section provides the detailed methodology of the work, focusing on both agent-based simulation and analytic approximation. The third section discusses results arising from simulations. This section also presents the comparative outcomes of simulations and the analytic approximation. Finally, last section delivers the concluding remarks of the work.

## Methods

In this section, we present two different approaches to explore the dynamic vaccination model, namely agent-based stochastic simulation, and a link percolation-based analytic approximation. In the following, we first provide a detailed illustration of the agent-based model (MAS) and then deliver an analytical approximation of the MAS model.

### Multi-agent simulation (MAS)

#### Disease spreading

We consider an infectious disease that propagates on a Barabási-Albert or BA scale-free network^24^ containing $N={10}^{4}$ nodes with an average degree $<k>=8$. The disease diffuses through the network following the SIR process in which a susceptible individual, having physical contact with an infected person, becomes infected with a rate $\beta$ (day^−1^ person^−1^). Also, the infected one recovers with the rate $\gamma$ (day^−1^). The model assumes that anyone who recovered from the disease remains in the recovered state forever. For the SIR process to be implemented in a time discretized MAS, we adopt the well-known Gillespie algorithm^33^, described by Fu et al. (see the supplementary material of^34^), to simulate the epidemiological process. To account for the effect of population structure, one needs to adjust the epidemic parameters^35^. In this regard, following the procedure of Fu et al.^34^ as well as other subsequent works (such as^36–38^) we choose the transmission rate $\beta$ such that the final epidemic size (FES), without vaccination, is that of the well-mixed population. The FES (${R}_{\infty }$) for the well-mixed population (without vaccination) satisfies^34^, ${R}_{\infty }=1-\mathrm{exp}(-{R}_{0}{R}_{\infty })$, where ${R}_{0}$ is the basic reproduction ratio. Taking ${R}_{0}=2.5$ and recovery rate $\gamma =1/3$ day^−1^ (the average infectious period is 3 days), we can estimate the FES for the well-mixed case as 0.8962 with the corresponding transmission rate for the scale-free network as, $\beta =0.196$ day^−1^ person^−1^. Note that we will use this $\beta$-value (corresponding to ${R}_{0}=2.5$) throughout the paper unless we state it explicitly.

#### Dynamic vaccination policy

**Figure 1:**
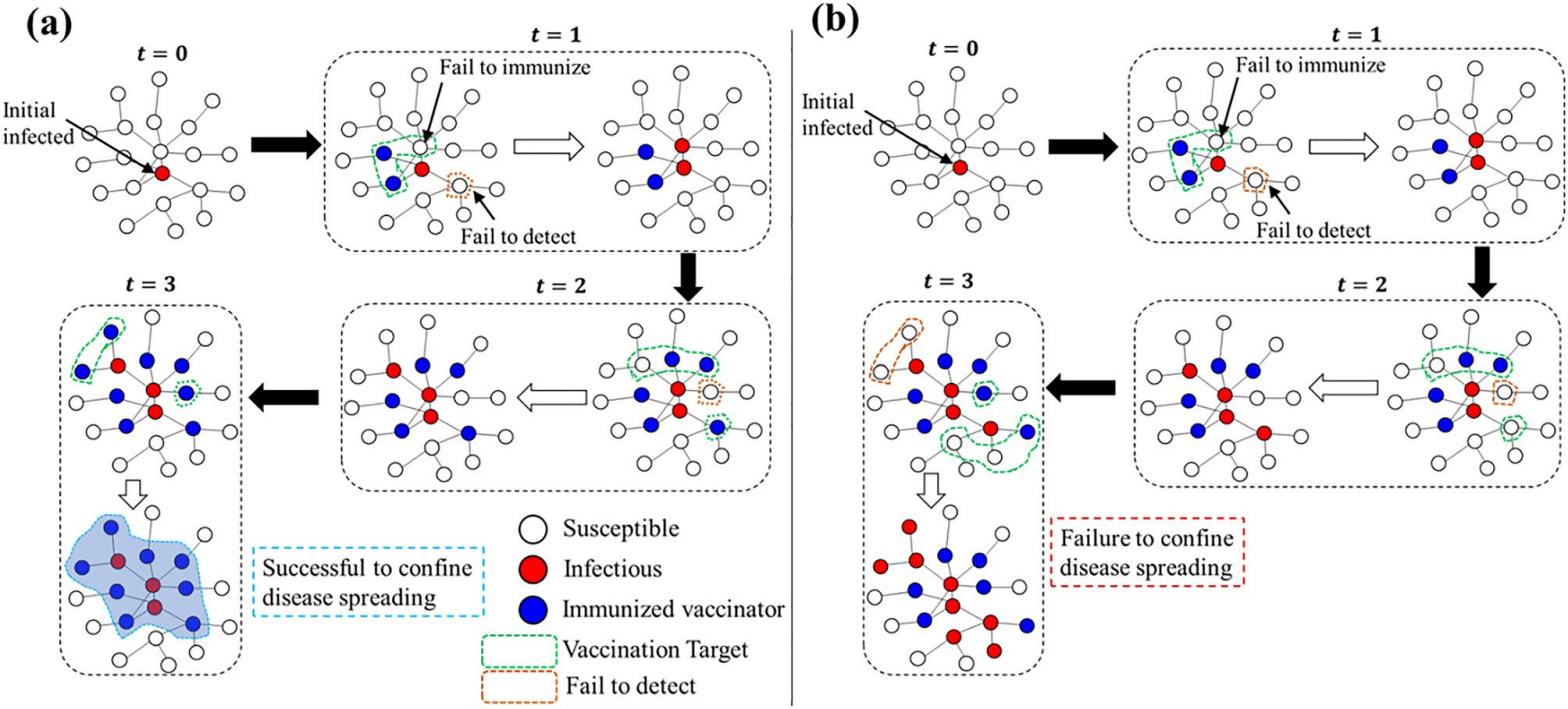
A schematic of the dynamic vaccination policy on a complex network. Here we portray two possible scenarios: success (panel **a**) or failure (panel **b**) to control the disease diffusion. The initial infection occurs at time $t=0$. The vaccination campaign starts in the next time step ($t=1$) by detecting susceptible individuals in the first neighborhood. However, all neighbors may not be traced due to information error (enclosed by orange-colored dashed line). Detected neighbors (enclosed by green dashed line) are given vaccination. A portion of the vaccinee fails to immunize due to vaccine failure. These issues may cause the infection to spread through the network. Depending upon the extent of vaccine efficacy and detecting error, there is a possibility of successful (panel **a**) or failed (panel **b**) confinement of the disease spreading.

The disease spreading starts with a random appearance of initially infected agents ${I}_{0}$ (we set ${I}_{0}=5$ throughout the work unless stated otherwise) on the network at time $t=0$. We assume that vaccines are available. The vaccination campaign starts at $t=1$, that is, one day after the onset of the epidemic. The first step is to identify susceptible people—staying in the first neighborhood of infected individuals—to have them vaccinated. This identification process is not always perfect, and the success or failure is determined by the influence of information noise as described below. The next step is to vaccinate the successfully detected susceptible individuals. Furthermore, as the vaccination is not perfect, a fraction of the vaccinee can still get infected due to vaccine failure. The same procedure will be implemented in the next time step. Note that the individual who has already been vaccinated is excluded from the vaccination target for the whole season. This process repeats until there is no infected individual in the system. Figure 1 demonstrates the schematic of the whole dynamical system, portraying two possible scenarios—success (panel (a)) or failure (panel (b)) to confine the disease spreading—which can occur as a possible consequence of detecting error and vaccine failure under the dynamic vaccination policy.

Since, in addition to the disease mitigation, our aim is to assess the overall social cost or social performance of the dynamic vaccination policy, at the end of the epidemic we evaluate each individual's gain based on her/his health status. Suppose the cost of vaccination and infection are denoted by ${C}_{v}$ and ${C}_{i}$, respectively with the condition ${C}_{v}\le {C}_{i}.$ Thus, the relative cost of vaccination (${C}_{r}$) is defined as, ${C}_{r}=\frac{{C}_{v}}{{C}_{i}}.$ Without loss of generality, we can set ${C}_{i}=1$, which enables us to consider ${C}_{v}\in \left[0,1\right]$, i.e., ${C}_{r}\in \left[\mathrm{0,1}\right].$ Hence a vaccinated person attaining perfect immunity (did not get infected), denoted by *HV*, incurs a vaccination cost ${C}_{r}$ (i.e., payoff: $-{C}_{r}$), whereas an infected yet vaccinated individual, denoted by *IV*, obtains a payoff of $-{C}_{r}-1$. A non-vaccinated but healthy person, denoted by *NH*, is associated with a payoff of 0, while an infected and non-vaccinated one, denoted by *NI*, is assigned a payoff of -1. Table 1 summarizes all possible gains according to the health status of individuals. Taking into account all possible payoffs, we finally estimate the average social payoff (ASP), as follows, to quantify the overall performance of the dynamic vaccination campaign for mitigating the disease spreading.

**Table 1:** Classification of individual’s gain according to the health status.

Vaccination/state	Healthy	Infected
Vaccinated	$-Cr$	$-Cr-1$
Not vaccinated	$0$	$-1$

1 $$ASP=-{C}_{r}*HV-\left({C}_{r}+1\right)*IV-NI=-{C}_{r}*VC-FES,$$

where $VC=(HV+IV)$ and $FES=(IV+NI)$ denote the vaccination coverage and final epidemic size, respectively. Note that a higher *ASP* indicates a lower social cost.

#### Vaccination efficacy and detecting error

We examine the impacts of two parameters—namely vaccine efficacy and detecting error—on whether the dynamic vaccination policy can successfully mitigate the disease spreading. As for vaccine efficacy, we assume that vaccine grants perfect immunity to a fraction $e\in [\mathrm{0,1}]$ (we name this parameter as ‘vaccine effectiveness’) of the vaccinators and no protection to the remainder. This is also called *failure-in-take* vaccine^39^. As for detecting errors, our model considers the following three types.

(i) Error type I $(E{r}_{I})$: An infected individual cannot be traced with the probability $E{r}_{I}.$ However, if the infected one is identified, all of her/his susceptible neighbors will surely be detected and vaccinated.
(ii) Error type II $(E{r}_{N})$: In this assumption, there is no failure to detect infected individuals. Nonetheless, the failure may occur in recognizing their susceptible neighbors with the probability $E{r}_{N}.$
(iii) Error type III $(E{r}_{I\&N})$: This case is a generalization of the above two errors. In contrast to error type II, this scenario takes into account the error in tracing infected individuals. If an infected person is detected, then—unlike error type I—some of the susceptible neighbors may not be detected for vaccination. Understandably, if the infected one is unnoticed, there is no question of searching for susceptible neighbors and consequently, they remain unidentified.

**Figure 2:**
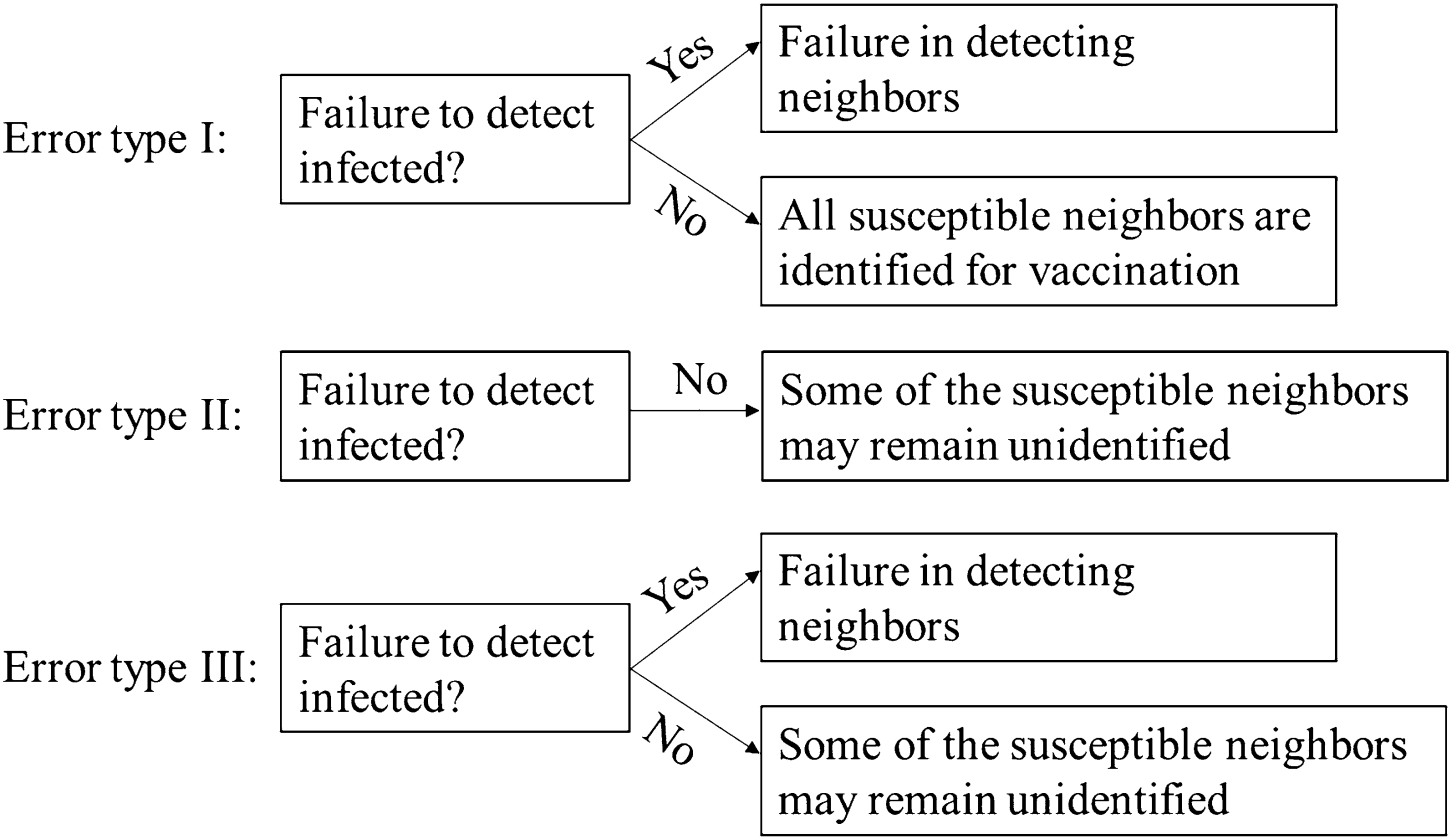
An illustration of three types of detecting errors. In error type I, the failure may occur in tracing infected ones, but not in detecting the susceptible neighbors of an identified infected person. In error type II, there is no failure in detecting infected individuals. The error can only occur in recognizing the susceptible neighbors. On the other hand, error type III combines both type I and II. A failure to trace the infected ones causes the failure in detecting the susceptible neighbors. Nonetheless, if the infected one is identified, some of the neighbors may remain unnoticed (unlike type I) for vaccination.

Clearly, error type III captures a more realistic scenario compared to the other two types (see Fig. 2). By assuming an error, for example, $E{r}_{I\&N}=0.5,$ we mean that an infected individual can be overlooked with the probability of 0.5; however, if the identification is successful, then each of the susceptible neighbors may fail to be detected with the probability of 0.5.

Taking into account the above factors, we run the agent-based simulation, following a similar procedure to that in^3,36–38^. The numerical results are obtained by taking an average over 100 independent episodes while changing the vaccine efficacy ($e$ or $\upeta$) and detecting errors ($E{r}_{I}$ or $E{r}_{N}$ or $E{r}_{I\&N}$) within the range from 0 up to 1.

### A link percolation-based analytic approximation model

Inspired by the work in Ref.^23^, here we present a link percolation^25,26,29,40^ model and use the generating function^27^ framework—taking into account imperfect vaccine (*failure-in-take vaccine*) and error in detecting susceptible neighbors—to approximate the results of stochastic simulations. Suppose a fraction $\omega$ of the susceptible neighbors, of an infected person, are being detected and thereby given vaccination. Consequently, the fraction $1-\omega$ (can be regarded as detecting error) remains undetected due to the lack of information. Furthermore, we assume that a fraction $e$ of the vaccinees acquires perfect immunity from vaccination. That is, the fraction of immunized vaccinee in the neighborhood of an infected node is $\omega e$, which indicates that the fraction $1-\omega e$ still remains susceptible. Hence, at each time step an infected agent infects a susceptible neighbor with the probability $\beta (1-\omega e)$ during a period of recovery time ${t}_{r}.$ Referring to^23,29^, we define the overall transmissibility ${T}_{\beta }\equiv {T}_{\beta }(\beta ,{t}_{r},\omega ,e)$ as the probability that a susceptible neighbor becomes infected before the focal infected individual recovers, which is given by the sum

2 $${T}_{\beta }=\left(1-\omega e\right)\beta {\Sigma }_{t=1}^{{t}_{r}}[\left(1-\omega e\right){\left(1-\beta \right)]}^{t-1}.$$

The term $\left(1-\omega e\right)(1-\beta )$ signifies the probability of remaining uninfected after each time step. In a similar way, we can define the transmissibility ${T}_{\omega }\equiv {T}_{\omega }(\beta ,{t}_{r},\omega )$ of vaccination which is the effective probability that a susceptible neighbor, of an infected agent, will be given vaccination within a period of time ${t}_{r}.$ This can be defined as^23^

3 $${T}_{\omega }=\omega {\Sigma }_{t=1}^{{t}_{r}}[\left(1-\omega \right){\left(1-\beta \right)]}^{t-1}.$$

Since the current work does not consider vaccinating behaviors—which can be elucidated by vaccination game models^34,41–47^ where a highly efficacious vaccine can increase the chance of vaccination—the above probability (Eq. (3)) is independent of vaccine efficacy.

The SIR process follows a treelike structure in which branches of infection develop and expand throughout the network^23^. Such a process can be mapped into link percolation^25,26^ which allows us to use the generating function framework. Suppose $f$ is the probability that a branch of infection will expand throughout the network^23^. The expression for $f$ satisfies the following transcendental equation

4 $$f=1-{G}_{1}\left(1-{T}_{\beta }f\right),$$

where ${G}_{1}={\sum }_{k={k}_{min} }^{{k}_{max}}\frac{kP\left(k\right)}{\langle k\rangle }{x}^{k-1}$,with $x\in \left[\mathrm{0,1}\right],$ is the generating function of the underlying branching process^23,25,26^.

Using above transmissibility parameters (Eq. (2–3)) and the expression for $f$ (Eq. (4)) in the generating function framework, we finally obtain (see Appendix B in SI for the detailed derivation) following equations for expressing the fractions of recovered (*R*, i.e., *FES*) and vaccinated (*V*) individuals, respectively

5 $$\begin{array}{c}R=\frac{{T}_{\beta }}{{T}_{\beta }+{eT}_{\omega }}\left(1-{G}_{0}\left(1-{T}_{\beta }f\right)\right),\end{array}$$

6 $$\begin{array}{c}VC=\frac{{T}_{\omega }}{{T}_{\beta }+{T}_{\omega }}\left(1-{G}_{0}\left(1-{T}_{\beta }f\right)\right),\end{array}$$

where $\frac{{T}_{\beta }}{{T}_{\beta }+{eT}_{\omega }}$ (or $\left(\frac{{T}_{\omega }}{{T}_{\beta }+{T}_{\omega }}\right)$) is the probability that a susceptible node becomes infected (or vaccinated) if its state is changed by one of its infected neighbors^23^. Also, ${G}_{0}={\sum }_{k={k}_{min} }^{{k}_{max}}P\left(k\right){x}^{k}$ is the generating function of the degree distribution $P(k)$^19,25,26^.

## Results and discussion

We choose model parameters focusing on an influenza type disease. However, it should be noted that the motivations and results of the model are not limited to influenza alone. It can be understood for other similar infectious diseases such as Covid-19. We summarize relevant parameter values in Table 2 which have been used throughout the simulations unless stated explicitly.

**Table 2:** Relevant parameters and their values used in multi-agent simulations.

Network	BA scale free^24^ with $\langle k\rangle =8$ and $N={10}^{4}$
Transmission rate $\beta$	$0.196$ day^−1^ person^−1^ corresponding to ${R}_{0}=2.5$^34^
Recovery rate $\gamma$	$1/3$ day^−1^^34^
Initial infection ${I}_{0}$	$5$(assumed)
Relative vaccination cost ${C}_{r}$	$0.5$(assumed)
Error in tracing infected individuals $E{r}_{I}$	[0,1]
Error in tracing susceptible neighbors $E{r}_{N}$	[0,1]
Error in tracing infected and susceptible neighbors $E{r}_{I\&N}$	[0,1]

These values have been presumed throughout unless stated otherwise.

### Impact of vaccine efficacy and detecting errors on dynamic vaccination

**Figure 3:**
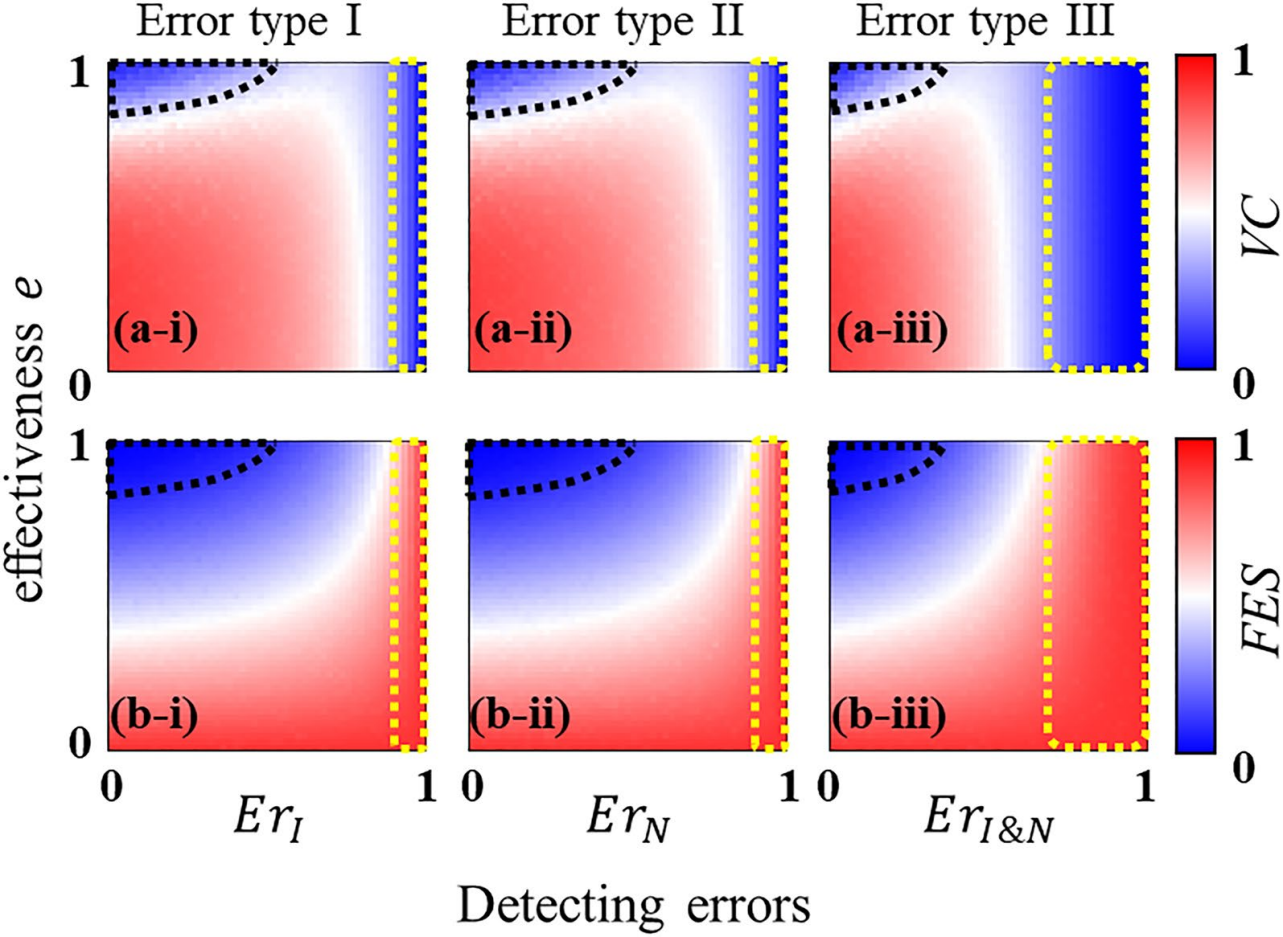
Stationary outcomes of the dynamic vaccination policy. Results are generated in terms of vaccination coverage (VC) and final epidemic size (FES) considering three error types. The dynamic vaccination can successfully mitigate the disease diffusion with a moderate to high level of vaccine effectiveness. A higher degree of detecting error causes a less vaccination coverage (blue region enclosed by yellow dotted line) and thereby, resulting in a higher disease prevalence (red region enclosed by yellow dotted line). Interestingly, a highly effective vaccine requires a less coverage (blue region enclosed by black dashed line in panel (**a**)) to attain a disease-free situation (corresponding blue region enclosed by black dashed line in panel (**b**)). Relevant parameter values are stated in Table 2.

Figure 3 demonstrates the outcomes of the dynamic vaccination campaign in terms of vaccination coverage (VC) and final epidemic size (FES) as a function of detecting error and vaccine efficacy in which we take into account three types of errors as discussed in “Vaccination efficacy and detecting error”. Results in panel (b) (Fig. 3) suggest that a moderate to a high level of effectiveness can ensure the disease suppression for a considerable detecting error. In the areas where the detecting errors (types I, II, and III) are quite high, the vaccine uptake level is quite low (blue regime enclosed by the yellow dotted line in panel (a) in Fig. 3), and thereby the dynamic vaccination campaign fails due to the high prevalence of the disease (red regime enclosed by yellow dotted line in panel (b) in Fig. 3). This means that due to the oversight of infectious persons and vaccination targets, the infection route cannot be blocked. Consequently, the disease emerges through the whole population. If the vaccine is less efficacious ($e$), then even a higher vaccine uptake (see the red region with lower effectiveness in panel (a) in Fig. 3) cannot guarantee the disease mitigation (a higher FES for a lower effectiveness in panel (b) in Fig. 3) which is quite conceivable. Contrarily, a highly efficacious vaccine requires less coverage (the blue region enclosed by the black dotted line in panel (a) in Fig. 3)—with a lower to moderate level of detecting error—to attain a disease-free situation (corresponding blue region enclosed by the black dotted line in panel (b) in Fig. 3). This is because an exceedingly efficacious vaccine can immediately cut off the infection route within a few time steps after detecting initially infected individuals (here we set ${I}_{0}=5$). This indicates that the initial confinement of the disease is possible only when vaccines are highly efficacious, and detecting errors are not so high. However, if there are many initial infections, for instance ${I}_{0}=500,$ that is, if the disease has already emerged with a greater degree, or if the disease transmissibility is quite strong, for example $\beta =0.6$ with ${R}_{0}=5$ (see Appendix C in SI), then the initial confinement of the disease may not be possible. Fig. C in Appendix C shows that in such a situation, the regime for the less disease prevalence, on the detecting errors versus vaccine efficacy heatmaps, shrinks compared to that in Fig. 3b. Moreover, it requires a larger vaccine uptake (even with a greater efficacy) and fewer detecting errors, to control the disease contagion. Understandably, error type III (i.e., $E{r}_{I\&N}$) imposes a greater possibility of disease diffusion than that of the other two types (i.e., $E{r}_{I}$ and $E{r}_{N}$). However, the patterns of the dynamics, arising from all error types, are almost comparable.

### Frequency of implementing vaccination

**Figure 4:**
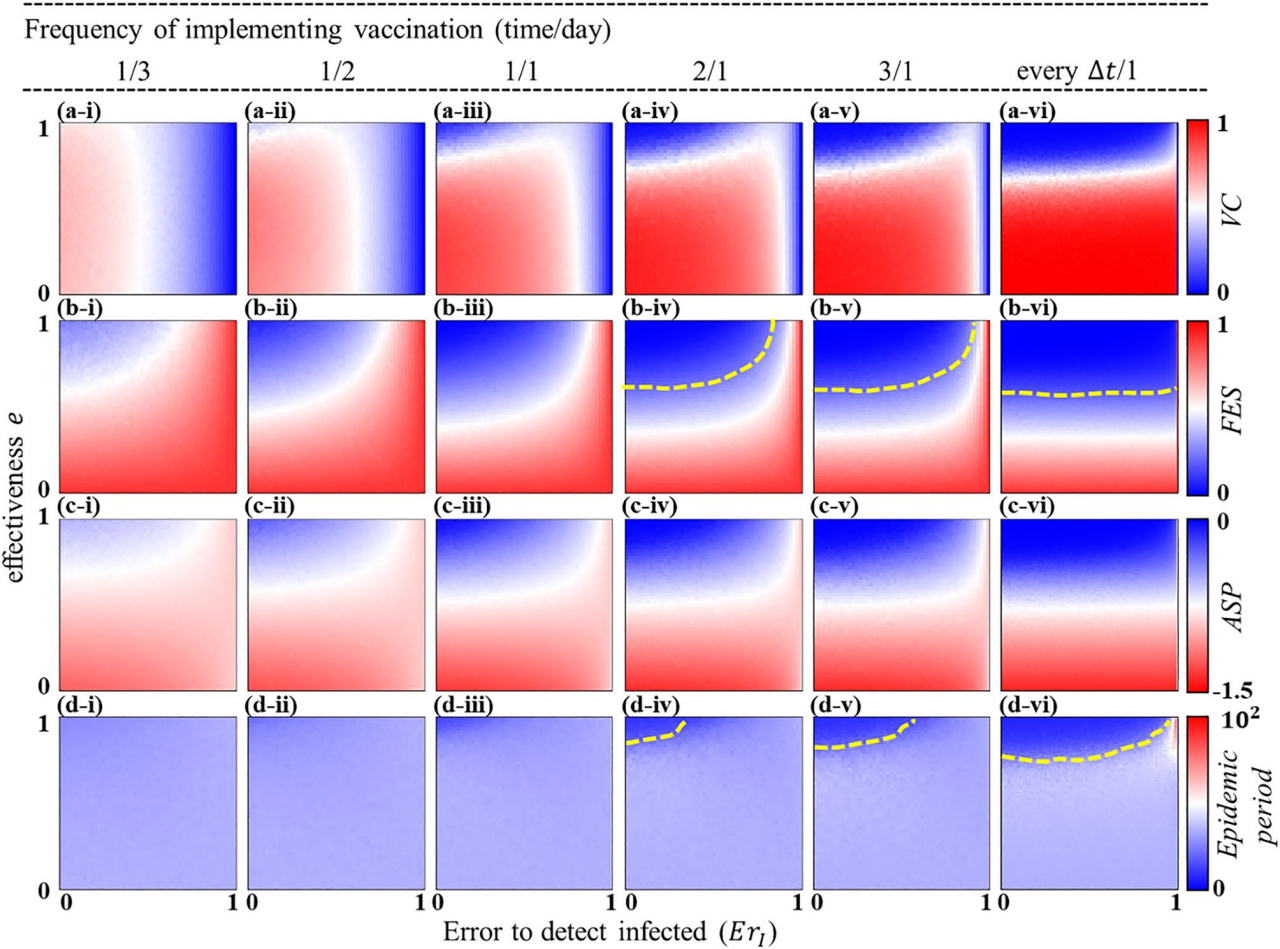
Impact of the frequency of vaccination campaign. This figure illustrates the performance of the dynamic vaccination, as a function of the detecting error (error in detecting infected node-$E{r}_{I}$) and vaccine efficacy, with increasing (from left to right) the frequency of screening for vaccination. Here we set the frequency as the number of times the screening process is conducted per day(s), i.e., time/day. The higher frequency in screening process upsurges the possibility of vaccination. Consequently, the dynamic vaccination can work well even with a higher detecting error (blue areas above yellow dotted lines in panels (**b, iv**–**vi**)). Also, the higher frequency of screening—with highly effective vaccine—reduces the time duration (blue regions above yellow dotted lines in panels (**d, iv**–**vi**)) of the disease prevalence. Note that the time step $\Delta t$ is estimated according to the Gillespie algorithm described in Appendix A of SI. Relevant parameter values are mentioned in Table 2.

So far, we have presumed that the screening for the vaccination is conducted once per day, that is, the vaccination is administered once in a day. We now intend to examine the performance of the dynamic vaccination by varying the frequency of the screening process. In Fig. 4, we investigate the efficiency of the dynamic vaccination by varying the frequency of screening from low to high. More specifically, we set the frequency as time/day. Panels (*-i) up to (*-vi) in Fig. 4 chronologically show the results—as a function of the detecting error (caused by the failure to trace infected ones) and vaccine effectiveness—when vaccination is implemented once per every three days (1/3), once per every two days (1/2), once per day (1/1), twice per day (2/1), thrice per day (3/1), and every $\Delta t$ (defined in Eq. (A2) in Appendix A of SI) time step per day, based on the Gillespie algorithm (described in Appendix A of SI). In particular, panel (*-vi) in Fig. 4 implies that the vaccination campaign is executed every time an agent changes its state, which represents the highest frequency of administering vaccination. The heatmaps from left to right demonstrate that the area of successful containment (blue regime in panels (a-*) and (b-*) of Fig. 4 where both *FES* and *VC* are low) expands as the frequency of vaccination surges. Interestingly, as the frequency of screening increases, the dynamic vaccination campaign can perform well even with a higher degree of detecting error (see blue regions above the yellow dotted lines in panels (b-iv) to (b-vi) in Fig. 4), especially when the vaccine is reasonably effective. This suggests that if the vaccination campaign is conducted more frequently, then—even though it is difficult to identify the vaccination targets—the chance for vaccination upsurges, which can eventually lead to a disease-free situation with an effective vaccine. This is why, we observe a relatively less sensitivity, along the detecting error, for the case of a highly frequent vaccination campaign (for instance, panel (*-vi) in Fig. 4). Contrarily, for a low effective vaccine, although *VC* is considerably high, *FES* is still large. This suggests that a less effective vaccine fails to control the epidemic even with a higher frequency of vaccination campaign (see red regions in panels (a-iv)–(a-vi) and (b-iv)–(b-vi) in Fig. 4). It is worth noting that the average social payoff (*ASP*), which accounts for the cost of infection and vaccination to assess the overall performance of the dynamic vaccination, seems to improve with the frequency of vaccination campaign (see blue regions in panels (c-i)–(c-vi) in Fig. 4). Furthermore, a higher frequency of screening—with a highly effective vaccine—can reduce the time required to end the disease prevalence (for instance, see blue areas above yellow dotted lines in Fig. 4d(iv–vi).

### Extending vaccination targets to the second neighborhood

**Figure 5:**
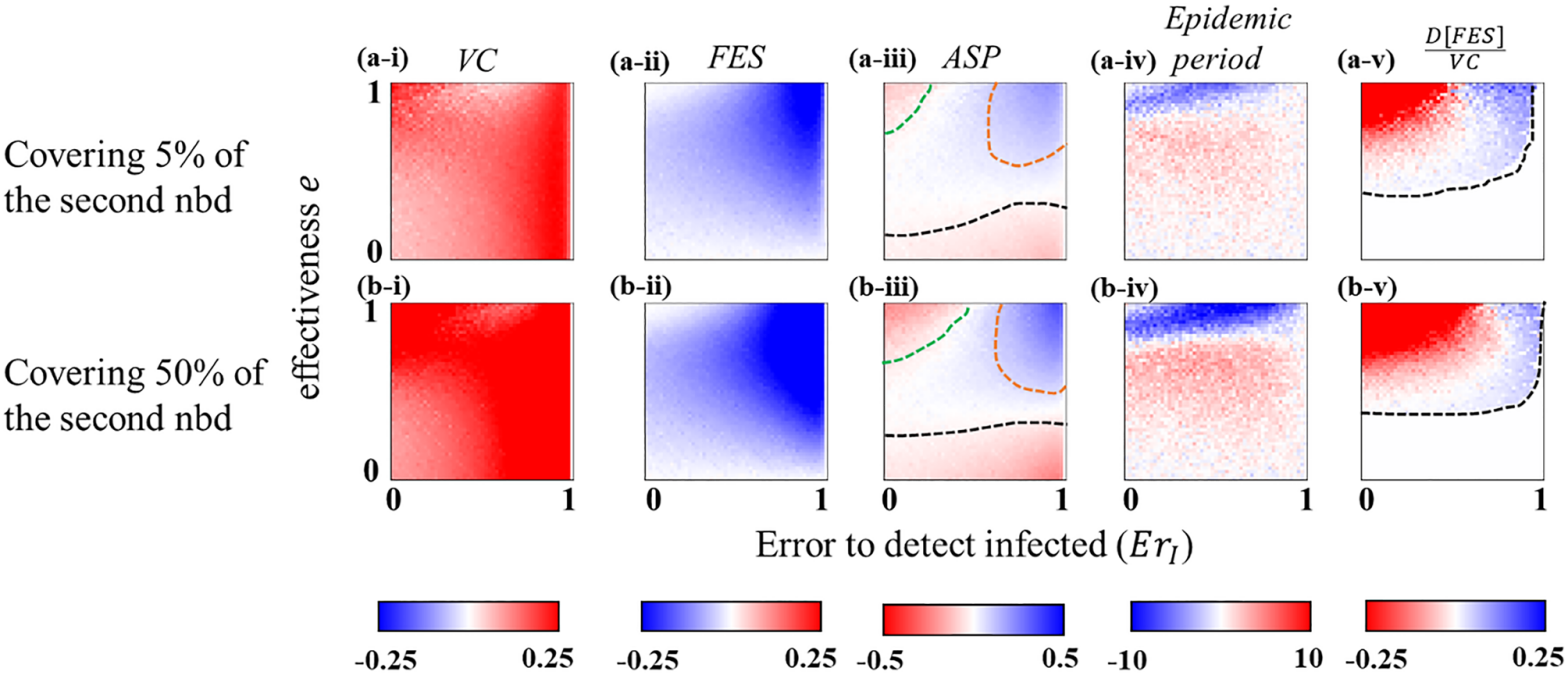
Expansion of the vaccination target. Results are obtained by subtracting outcomes focusing only on the first neighborhood as the vaccination target (i.e., the default case as in Fig. 3a,b) from that of the expansion to the second neighborhood (covering 5% (upper panel) and 50% (lower panel)). Quantity of interests, from left to right, chronologically depict vaccination coverage (a-*), final epidemic size (b-*), average social payoff (c-*), total time until the end of the disease prevalence (d-*), and (**e**) the efficiency in reducing *FES* by a single vaccination dose. In the right most column, we define $D[FES]/VC=(FE{S}_{VC=0}-FES)/VC.$ In the case of *ASP* (panel (*-iii)), blue (red) stands for a better(worse) outcome in the extended range of vaccination targets. Obviously, it would require more vaccination coverage (yielding less FES) in the case of extended vaccination targets. With regard to the overall social gain (*ASP* defined in Eq. (1)), it would be beneficial to extend vaccination targets to the second neighborhood when vaccines are highly effective, and the detecting error is quite large (blue region enclosed by orange dotted line panel (*-iii)). The expansion to the second neighborhood would require less time to end the disease prevalence only when vaccines are highly efficacious (blue region in panel (*-iv)). The black dotted line in panels (*-v) depicts the boundary above which $FES\le 0.45$. Below this line, the dynamic vaccination fails to confine the diseases spreading. Relevant parameters values are stated in Table 2.

We now investigate whether—in addition to the first neighborhood—extending vaccination targets to the second neighborhood of an infected agent (if successfully detected) can outperform the default case (i.e., focusing on the first neighborhood only). The aim is to examine whether such an extension can improve the epidemic scenario when vaccines are less efficacious, or the detecting errors are quite high. Since it is practically difficult to trace out all individuals in the second neighborhood, we examine two cases: taking into account 5% (Fig. 5a-*) and 50% (Fig. 5b-*) of the second neighborhood (randomly) of an identified infected individual as the vaccination target. Note that in this case, we only consider the error in detecting infected individuals ($E{r}_{I}$) as the other two error types possess almost similar dynamics. Figure 5 illustrates the results by subtracting outcomes focusing only on the first neighborhood (i.e., the default case as in Fig. 3a,b) from that of the expansion to the second neighborhood (5% or 50%). Our discussion in this regard will be based on the comparative performance, quantified by the average social payoff (*ASP*), of both cases. The results indicate that the difference in *ASP* in Fig. 5(*-iii)—where the blue (red) color delineates a better (worse) *ASP* than the default case—varies over the detecting error ($E{r}_{I}$) vs. effectiveness heatmap although *VC* (*FES*) seems larger (smaller) in the extended neighborhood version (see panels (*-i) and (*-ii) in Fig. 5). We provide reasoning for such variations in *ASP* for three different scenarios. (i) Highly effective vaccine and low detecting error: In this case, *ASP* for the extended neighborhood is worse than the default case (red region in the upper left corner enclosed by green dotted line in panels (*-iii) in Fig. 5). In such a situation, *FES* in the default case (see Fig. 3b-*) is sufficiently low due to a highly effective vaccine with less detecting error, and thereby, extending the vaccination range requires ‘extra’ vaccinations, which accordingly worsen the *ASP*. Furthermore, $D[FES]/VC=(FE{S}_{VC=0}-FES)/VC$(here $FE{S}_{VC=0}$ means the *FES* without vaccination), which quantifies the effect of reducing FES by a single vaccination dose, has deteriorated (red regions in Fig. 5*-v). This illustrates that it would not be worthy (concerning the social cost) of expanding vaccination targets if vaccines are highly effective, and infected people are being detected with less error. (ii) Less vaccine efficacy: The extended neighborhood case again yields less *ASP* than the default case (red region below the black dotted line in Fig. 5*-iii). In such a situation, due to inefficacious vaccines, the infection route cannot be cut off, even with expanding the vaccination range. Hence, extending vaccination targets to the second neighborhood would not be beneficial for this scenario. (iii) High $e$ and high error: The expansion of vaccination targets now yields a better *ASP* than the default case (blue region enclosed by orange dotted line in Fig. 5*-iii). With highly effective vaccines, the expansion of the vaccination range makes it possible to reduce the infection spreading, even under the condition of a high detecting error ($E{r}_{I}$). As a result, the number of infected people is decreased significantly (see corresponding blue regions in Fig. 5*-ii). Additionally, $D[FES]/VC$ becomes better, that is, the vaccination efficiency in reducing *FES* upsurges (see the corresponding region in Fig. 5*-v). Thus, in this situation, it would be advantageous to expand vaccination targets to the second nearest neighbors, even with a high detecting error. Regarding the time until the end of the disease prevalence, extending vaccination targets requires less time to end the disease spreading only when vaccines are highly effective (see the blue region in Fig. 5*-iv).

**Figure 6:**
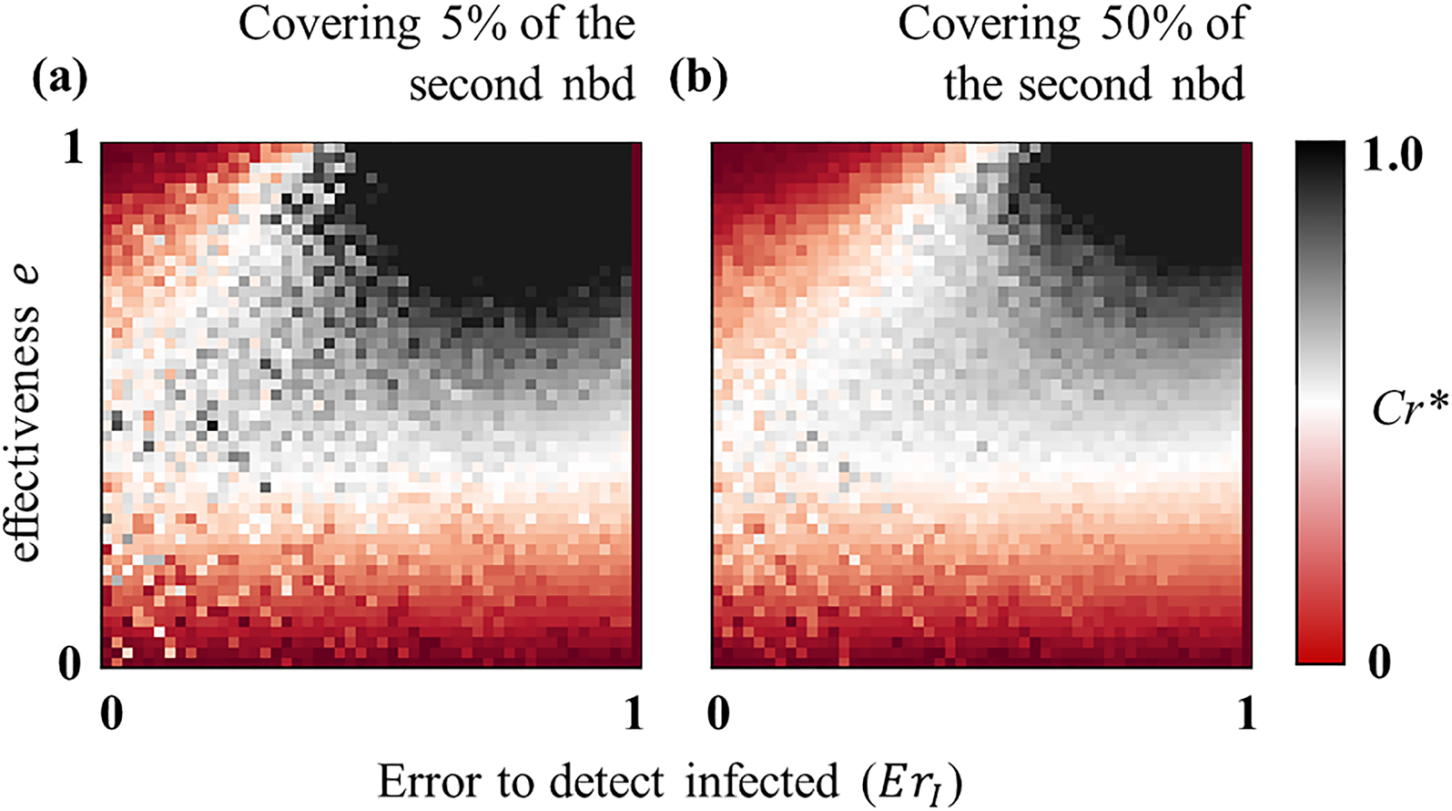
Examining the social benefit regarding the expansion of the vaccination target. The critical vaccination cost $C{r}^{*}$, as a function of detecting error and *e*, at which the *ASP* estimated in the expansion (to the second neighborhood) strategy (for vaccination) is equivalent to that in the default case (covering only the first neighborhood). Expanding vaccination targets makes the total social cost lower than the default case whenever $Cr<C{r}^{*}$. ${C}_{r}^{*}\approx 1$ in the black regimes in both panels which demonstrates that expansion strategy can reduce the overall social cost in those scenarios. On the other hand, the red regions (in both panels) illustrate that ${C}_{r}^{*}\approx 0$, which means that it would not be beneficial to expand vaccination targets in those cases as ${C}_{r}\ge 0.$ Relevant parameters values are stated in Table 2.

Furthermore, Fig. 6 demonstrates the critical relative vaccination cost $C{r}^{*}$ at which *ASP* obtained in the expansion strategy is equal to that in the default case, meaning that adopting the expansion strategy would be more beneficial for the society whenever $Cr$ is less than $C{r}^{*}$. Corresponding to the scenarios (i) and (ii) described above, Fig. 6 shows that ${C}_{r}^{*}$ is almost zero (red region in panels (a, b) in Fig. 6) which indicates that the expansion strategy always increases the social cost no matter how cheap the vaccination cost is. In contrast, the advantageous situation is scenario (iii), where $C{r}^{*}$ is almost 1.0, suggesting that the social cost can be minimized by expanding the vaccination range, regardless of the cost $Cr(<{C}_{r}^{*}\approx 1)$.

### Stochastic simulation versus analytic approximation

**Figure 7:**
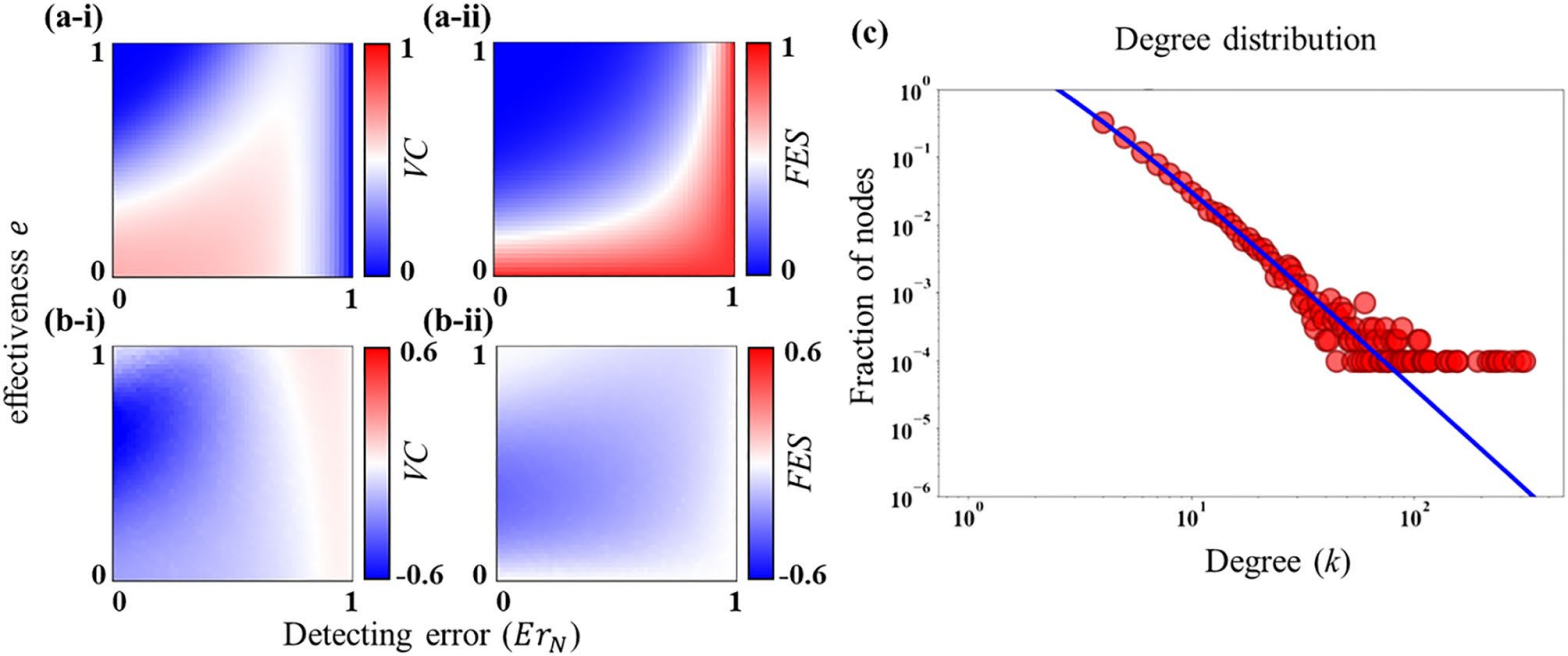
Results obtained from the analytic method. Outcomes are generated from the analytic approximation (illustrated in Eqs. (2)–(6)) as a function of detecting error (error in detecting susceptible neighbors, $E{r}_{N}$) and vaccine efficacy $e$ (panels (a-*)). Panel (b-*) demonstrates the difference in outcomes by subtracting results obtained in MAS (i.e., the corresponding panels (a-ii) and (b-ii) in Fig. 3) from that of the analytic approximation. The gap between the analytical prediction and stochastic simulation arises from the deviation in degree distribution (for BA scale-free network with $<k>=8$) between theory and MAS. Note that the analytic approximation assumes the degree distribution as $P\left(k\right)= \frac{2m\left(m+1\right)}{k\left(k+1\right)\left(k+2\right)},$ where $m$ is the half of the average degree^48^. Furthermore, we adjust the transmission rate $\beta$ for the analytic method as $\beta =0.148$ (see Appendix D in SI for more details). Other relevant parameters are stated in Table 2.

We finally compare results obtained from the analytic approximation (link-percolation model demonstrated in “A link percolation-based analytic approximation model”) and agent-based stochastic simulations (i.e., MAS). Note that the analytic approximation only captures the scenario with the error in detecting susceptible neighbors (i.e., $E{r}_{N}$). Panels (a-i) and (a-ii) of Fig. 7 illustrate analytic approximations, in terms of *VC* and *FES*, of the stochastic simulations presented in panels (a-ii) and (b-ii) of Fig. 3. Comparing both results, we can perceive that the pattern of the dynamics in the analytic approximations is almost similar compared to the stochastic simulation albeit having some discrepancies. Panels (b-i) and (b-ii) in Fig. 7 depict such discrepancies by subtracting the results of stochastic simulations from that of the theoretical approximation. Overall, the theoretical prediction yields less *FES* than the simulation, which is caused by the difference in degree distribution between theory and the actual scenario (see Fig. 7c). Following Ref.^48^, we choose the degree distribution for the scale-free network as $P\left(k\right)= \frac{2m\left(m+1\right)}{k\left(k+1\right)\left(k+2\right)},$ where $m$ is the half of the average degree. Also, in the generating function (i.e., ${G}_{0}$ defined in Sect. 2.2) of the degree distribution, we set ${k}_{min}=4$ and ${k}_{max}=312$ to agree with the actual network configuration used in the simulation. Furthermore, the transmission rate $\beta$ for the analytic approximation is estimated as 0.148 so that *FES* in the approximation framework, without vaccination, is that of the well-mixed population (details are given in Appendix D of SI). Compared to MAS, the approximation framework has a smaller proportion of Hub agents, especially nodes having degrees more than 100 (Fig. 7c). Hence, there is a relatively less chance of epidemic propagation from the analytic approximation. In addition, Fig. 7b–i reveals that the analytic approximation generally exhibits less vaccination coverage than MAS except for the case with high detecting error. The vaccination coverage depends upon the extent of detecting error and the total vaccination target. Since the approximation framework possesses fewer vaccination targets than that of MAS, with the same detecting error for both methods, the total number of vaccinators, per a single vaccination campaign, in the approximation framework is always smaller than MAS. Consequently, the former consumes less vaccination coverage as a whole. In contrast, in a high detecting error case, the identification of vaccination targets is hampered, to a greater scale in MAS, due to the presence of super-hub agents (i.e., agents having an extremely high degree). Since the impact brought by hub agents can be more significant in simulations, the total vaccination opportunities in MAS becomes lower than the analytic method.

## Conclusion

Fighting against an epidemic or a pandemic requires strategic policymaking that can mitigate the disease spreading taking into account various practical hindrances. The current Covid-19 pandemic has caused us to experience several obstacles—such as vaccine scarcity, vaccine efficacy, unconsciousness—in containing the infection spreading. The current work particularly takes into consideration some of these barriers and designs a dynamic vaccination strategy where susceptible neighbors of infected individuals are detected and given vaccination before having any physical contact. Our purpose was to investigate whether such a policy can successfully cut off the infection route of disease while minimizing the overall social cost. Detecting infected as well as their susceptible neighbors cannot be flawless. Moreover, vaccines may not confer perfect immunity. Our model considers these pragmatic issues to demonstrate a more realistic picture.

It is worth stressing that although the model parameters (Table 2) are chosen focusing on an influenza type disease, the results are not limited to influenza alone. It can be understood within the context of other infectious diseases, such as Covid-19, as well since the motivation and background are almost similar. Our simulation reveals that the dynamic vaccination campaign can successfully diminish the disease diffusion as long as vaccines grant a moderate to high efficacy with a reasonable detecting error (Fig. 3a,b). In general, each error type exhibits almost a similar pattern of the dynamics although the degree of the impact caused by each type can be different. However, some of the findings could be affected by the change of parameters. For example, it is observed according to the parameter choice in Table 2 that if vaccines are highly efficacious, and the detecting error is low, it is possible to confine the disease spreading—by administering a less amount of vaccination—within a short period (Fig. 3a,b). Nonetheless, this phenomenon may not be possible under the condition of a stronger disease transmissibility, which can be quantified by a higher basic reproduction number, say ${R}_{0}=5$ (see Appendix C in SI). We have found that in such a situation, the efficiency of the vaccination strategy in mitigating infections reduces significantly (see panel (d) in Fig. C in SI). Another aspect to be noted is that we have presumed the same recovery rate ($\gamma =1/3$) throughout the work (for simplicity). It is, however, conceivable that increasing the recovery rate, while keeping the transmission probability fixed, would decrease the epidemic threshold ${R}_{0}(=\beta /\gamma )$, and consequently, reduce the epidemic size. In such a scenario, the dynamic vaccination will perform even better than the current parameter setting.

As errors in detecting infected or susceptible neighbors cannot be entirely avoided in practice, we resort to remedy this issue indirectly by varying the frequency of employing vaccination and expanding vaccination targets from the first neighborhood up to a certain range of the second neighborhood. The chance for vaccination upsurges (thus, *FES* decreases) with the increase of the frequency of conducting vaccination campaigns, which accordingly reduces the impact of the error in detecting susceptible neighbors (Fig. 4). Such a situation inevitably grants a better average societal payoff (i.e., *ASP* quantified by the extent of *VC* and *FES* with associated costs); in other words, reduces the overall social cost. Furthermore, the expansion of the vaccination targets (of an infected person) has been found to be economically beneficial for society only when vaccines are effective, but the error in tracing susceptible neighbors is quite high (Fig. 5*-iii). Our results further suggest that expanding vaccination targets would not be worthwhile for a highly effective vaccine alongside a less detecting error (in identifying neighbors) because in such a case, focusing only on the first neighborhood—for vaccination—can efficiently diminish the disease spreading.

Finally, inspired by the work in^23^, we have presented an analytic approximation of the model using a link percolation-based generating function framework. This approach can greatly reduce the computational cost associated with agent-based stochastic simulations. In general, the analytic approximation provides an almost similar pattern of the dynamics—about detecting error (to find neighbors) and vaccine efficacy—compared to that in MAS (multi-agent simulation) despite showing some discrepancies. We argue that such discrepancies arise due to the deviation in degree distribution between theory and simulation (Fig. 7c). More specifically, the actual network configuration in MAS contains more hub agents compared to the theory and accordingly, leads to a higher epidemic size (*FES*) than the analytical method.

The concept of dynamic vaccination in the current work postulates that an individual immediately obtains immunity right after an effective vaccination is administered, which can be regarded as an idealized assumption since, in reality, there is a certain time delay to start the immune system to work after vaccination. The inclusion of such a premise may demonstrate a more pragmatic context. Furthermore, all our analyses have been illustrated based on the scale-free network. However, the investigation on other networks (such as small-world^49^) could provide a more general impression of the current work. In addition, the work disregards the notion of human behaviors towards vaccination. However, in reality, behavioral attitude can alter outbreak trajectories of infectious diseases^50,51^. Therefore, it would be interesting to explore the inclusion of the behavioral aspect towards vaccination in the current context. More specifically, as the link percolation-based generating function framework can predict the steady-state behavior of the system in structured populations^23^, one fascinating extension could be the introduction of vaccinating behavioral dynamics (which is well depicted by vaccination game^34,41–47^) into that analytical framework.

## Supplementary Information

Supplementary Information.
