# Supplementary material for: Investigating the efficiency of dynamic vaccination by consolidating detecting errors and vaccine efficacy
Source: Sci Rep. 2022 May 17;12:8111. doi: 10.1038/s41598-022-12039-1 (PMC9114144; doi:10.1038/s41598-022-12039-1)
Supplement: Supplementary file 1 — Supplementary Information. [file 41598_2022_12039_MOESM1_ESM.docx]

Supplementary information

Investigating the efficiency of dynamic vaccination by consolidating detecting errors and vaccine efficacy

**Author names and affiliations**

Yuichi Tatsukawa^a*^, Md. Rajib Arefin^a,b*^, Shinobu Utsumi^a^, Jun Tanimoto^a,c^

^a^Interdisciplinary Graduate School of Engineering Sciences, Kyushu University, Kasuga-koen, Kasuga-shi, Fukuoka 816-8580, Japan

^b^Department of Mathematics, University of Dhaka, Dhaka-1000, Bangladesh

^c^Faculty of Engineering Sciences, Kyushu University, Kasuga-koen, Kasuga-shi, Fukuoka 816-8580, Japan

***Corresponding author**

[tatsukawa.yuichi.534@s.kyushu-u.ac.jp](mailto:tatsukawa.yuichi.534@s.kyushu-u.ac.jp) (Y. T.), [arefin.math@du.ac.bd](mailto:arefin.math@du.ac.bd) (M. R. A.)

**Appendix A**: Gillespie algorithm for simulating the epidemic process

Following Ref.^1^, here we use the Gillespie algorithm ^2^ to simulate the disease propagation process in structured populations.

1. We calculate transition probabilities of all individuals (susceptible and infected) that has a possibility of state transition at a certain time t [day]. The transmission probability for a susceptible person is $p_{i}\left( t \right)=\beta\times$ number of infected neighbors, and that of an infected one is $p_{i}\left( t \right)=\gamma$. Thus, total probability of whole population is represented by
2. Next, calculate the time $t^{'}$ at which the next transition event occurs, i.e., $t^{'}=t+\Delta t,$ where $\Delta t$ is sampled from an exponential distribution with mean $\frac{1}{\lambda\left( t \right)}$. Thus, $\Delta t$ is given by

where $u\in[0,1)$ is a uniform random number.

1. Finally, select an individual that will change her/his state at the next timestep. This process needs to generate another uniform random number *v* ($v\in[0,1)$). One individual *k* is determined from candidates obeying to the following condition

Note that $\sum_{j=1}^{0} \frac{p_{j}\left( t \right)}{\lambda\left( t \right)}=0.$

1. Repeat steps (i)-(iii) until there is no infected individuals in the population or stop after a predefined time duration.

**Appendix B:** Derivation of the link percolation-based analytic model

As mentioned in the body text that this analytic approximation model is formulated following the previous work in Ref ^3^. In the main text, we have already shown the overall disease transmissibility $T_{\beta}\equiv T_{\beta}(\beta,t_{r},\omega,e)$ as follows

$T_{\beta}=\left( 1-\omega e \right)\beta\Sigma_{t=1}^{t_{r}}[\left( 1-\omega e \right){\left( 1-\beta\right)]}^{t-1}, (B1)$

where $\omega$ is the fraction of susceptible who are successfully detected to give vaccination. We also define the overall transmissibility $T_{\omega}\equiv T_{\omega}(\beta,t_{r},\omega)$ as the probability that a susceptible neighbor, of an infected person, becomes vaccinated. This probability has been defined as

$T_{\omega}=\omega\Sigma_{t=1}^{t_{r}}[\left( 1-\omega\right){\left( 1-\beta\right)]}^{t-1}. (B2)$

The SIR model follows a treelike structure where branches of infections develop and expand throughout the network, and the infection can only move forwards since the reinfection is assumed impossible ^3^. Such a system can be mapped into link percolation ^4,5^ which enables us to use the generating function framework to illustrate the dynamic. If $f$ is the probability that a branch of infection reaches a node, with a degree $k$, across a randomly chosen connection, and it expands via remaining $k-1$ links, then$f$ satisfies the transcendental equation $\begin{aligned} f=1-G_{1}\left( 1-Tf \right) (B3) \end{aligned}$

where $T$ is the disease transmissibility (i.e., probability of infection), and $G_{1}=\sum_{k=k_{min}}^{k_{max}} \frac{kP\left( k \right)}{\left\langle k \right\rangle}x^{k-1}$,with $x\in\left[ 0,1 \right],$ is the generating function of the underlying branching process ^3–5^. It is worth noting that $G_{1}(1-Tf)$ indicates the probability that branches of infection do not extend across the network ^3^. According to link percolation theory, if the transmissibility $T<T_{c}$, where $T_{c}$ is a critical threshold of the process separating epidemic and nonepidemic phases at steady state, then there is an epidemic free phase, which corresponds to finite clusters. On the other hand, $T>T_{c}$ indicates an epidemic phase in which branches of infection contribute to a spanning cluster of recovered people ^3,5^. Therefore, the probability to randomly select a node that belongs to the spanning cluster of recovered individuals ($R$) can be written as

$\begin{aligned} R=1-G_{0}\left( 1-Tf \right), (B4) \end{aligned}$where $G_{0}=\sum_{k=k_{min}}^{k_{max}} P\left( k \right)x^{k}$ is the generating function of the degree distribution $P(k)$ ^3–5^.

On the course of expanding the branches of infection, the infection branch reaches both recovered and vaccinated nodes. Therefore, in the steady state, the sum of the fraction of recovered (*R*) and vaccinated (*V*) individuals maps with the order parameter of link percolation ^3^. A susceptible node can become infected or vaccinated if it is connected to an infected agent via a S-I link. Thus, it is necessary to take into account the probability that a susceptible node becomes infected or vaccinated if it has at least one infected neighbor. When the susceptible node becomes infected, this probability can be estimated as $\frac{T_{\beta}}{T_{\beta}+{eT}_{\omega}}$, whereas if the susceptible becomes vaccinated, such a probability can be taken as $\frac{T_{\omega}}{T_{\beta}+T_{\omega}}$. It is important to note that since—unlike the probability that a susceptible becomes infected—the probability of becoming vaccinated does not depend on the vaccine efficacy $e$, the latter probability is independent of $e$. Hence, for the FES or recovered fraction *R*, we consider the probability that a randomly selected node is connected to a branch of infection via at least one of its $k$ connections ^3^. Then using Eqs. (B1-B4), FES is written as

In the same way, the fraction of vaccinated nodes can be written as,

**Appendix C:** Outcomes of the dynamic vaccination under a higher number of initial infections and a greater $R_{0}$


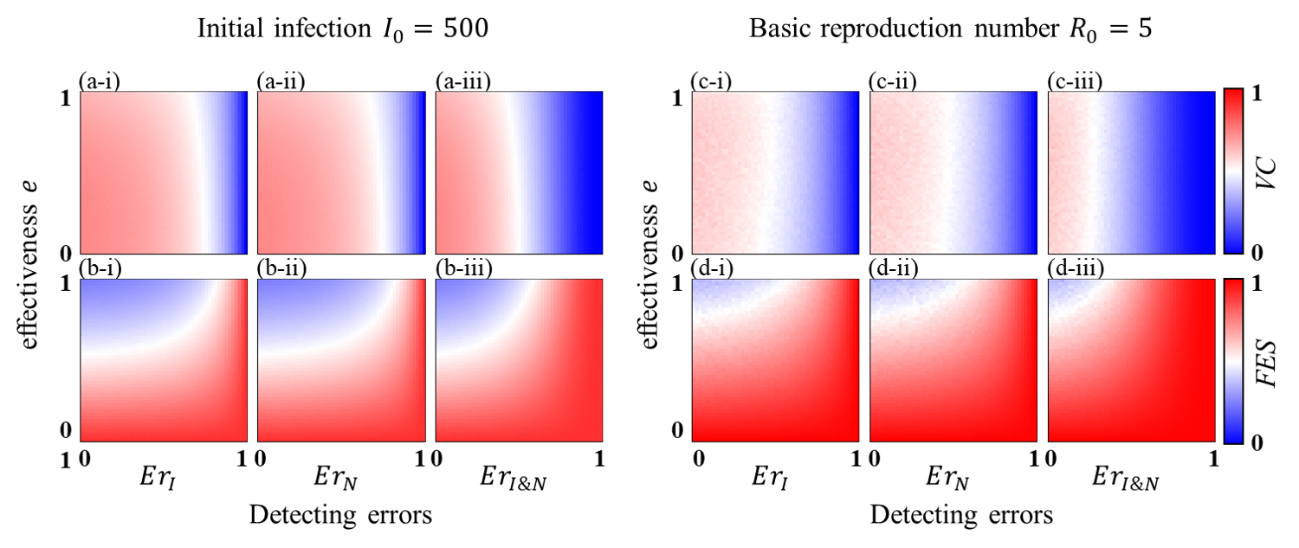


Fig. C: Outcomes of the dynamic vaccination policy as a function of detecting error and vaccine efficacy with a higher initial infection (panels (a, b)) and a larger basic reproduction ratio $R_{0}$ (panels (c, d)). Panels (a, b) show the instance when the disease has already emerged before the dynamic vaccination campaign starts, whereas panels (c, d) represent the scenario of a stronger disease transmissibility. Panels (d-*) demonstrate the difficulty to confine the disease diffusion by the dynamic vaccination policy under a larger $R_{0}.$ Note that the transmission probability $\beta$ in panels (c, d) is estimated as 0.6 (following Ref. ^1^) to agree with $R_{0}=5.$ Moreover, $I_{0}$ in panels (c, d) is taken as 5, whereas $R_{0}$ in panels (a, b) is chosen as 2.5.

Here, we discuss how DV performs if the disease has already emerged through the population (which can be illustrated by the higher number of initial infections) to a greater extent, or if the disease spreads with a stronger intensity (which can be demonstrated by a higher $R_{0}$ value). Fig. C(a, b) represents the instance of a higher initial infection (say, $I_{0}=500$ with $R_{0}=2.5$), and Fig. C(c, d) illustrates the outcomes for a greater basic reproduction number $R_{0}$ (we choose $R_{0}=5$ with a corresponding $\beta=0.6$). In both cases, FES increases, and the area of successful containment of the disease shrinks compared to default condition in Fig. 3(b). Additionally, the sensitivity along the vaccine effectiveness $e$ with respect to vaccination coverage is extremely small. This is constructed by two aspects. In the region of large $e$, the vaccine uptake level required to control the disease transmission increases (comparing to the default case in Fig. 3(a)), whereas in the region of small $e$, an acceleration of disease spreading causes a severe epidemic before the dynamic vaccination campaign performs, resulting in a reduction of the total number of vaccine doses.

**Appendix D:** Estimation of $\beta$ for the link-percolation based analytic method

To agree with the stochastic simulation, we adjust the transmission rate $\beta$ for the approximation method, following the similar procedure presented in Ref. ^1^. The idea is to choose a $\beta$ (here we denote it by $\beta^{*}$) such that FES—without vaccination—estimated from the approximation method is that of the well-mixed situation (without vaccination). As we have already mentioned in the body text (section 2.1.1) that the final epidemic size (*FES,* $R_{\infty}$), corresponding to $R_{0}=2.5$ (basic reproduction number), in the well-mixed population (without vaccination) is estimated as $R_{\infty}=0.899$. The corresponding $\beta$ for such *FES* is evaluated by generating $R_{\infty}$ (without vaccination), from the approximation method (i.e., Eq. (5) in the body text), as a function of $\beta$. The value of $\beta$ at which $R_{\infty}=0.899$ is the corresponding transmission rate ($\beta^{*}$), which is estimated as $\beta^{*}=0.148$ (see Fig. D).


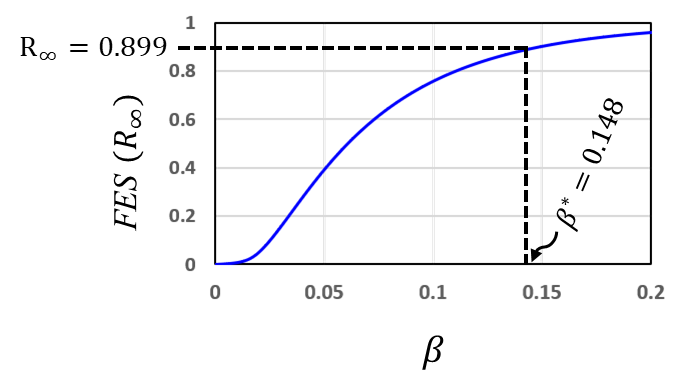


Fig. D: The final epidemic size (FES, $R_{\infty}$)—evaluated from the link-percolation based analytic method described in section 2.2—is depicted as a function of the transmission rate $\beta$ without vaccination. The required $\beta$ (i.e., $\beta^{*}$) is determined by choosing the transmission rate that corresponds to $R_{\infty}=0.899$ (*FES* estimated from the well-mixed population for $R_{0}=2.5$).

References

1. Fu, F., Rosenbloom, D. I., Wang, L. & Nowak, M. A. Imitation dynamics of vaccination behaviour on social networks. *Proc. R. Soc. B* **278**, 42–49 (2011).

2. Gillespie, D. T. Exact stochastic simulation of coupled chemical reactions. in *Journal of Physical Chemistry* **81**, 2340–2361 (American Chemical Society, 1977).

3. Alvarez-Zuzek, L. G., Di Muro, M. A., Halvin, S. & Braunstein, L. A. Dynamic vaccination in partially overlapped multiplex network. *Phys. Rev. E* **99**, 1–11 (2010).

4. Newman, M. E. J., Strogatz, S. H. & Watts, D. J. Random graphs with arbitrary degree distributions and their applications. *Phys. Rev. E* **64**, 026118 (2001).

5. Braunstein, L. A. *et al.* *Optimal path and minimal spanning trees in random weighted networks*. *International Journal of Bifurcation and Chaos* **17**, (World Scientific Publishing Company, 2007).
